# Role of NFAT5 in Inflammatory Disorders Associated with Osmotic Stress

**DOI:** 10.2174/138920210793360961

**Published:** 2010-12

**Authors:** Wolfgang Neuhofer

**Affiliations:** Departments of Nephrology and Physiology, Inner City Campus, University of Munich, Munich, Germany

**Keywords:** NFAT5, osmotic stress, cytokines, inflammation, gene regulation.

## Abstract

Nuclear factor of activated T cells 5 (NFAT5) is the most recently described member of the *Rel* family of transcription factors, including NF-κB and NFAT1-4, which play central roles in inducible gene expression during the immune response. NFAT5 was initially described to drive osmoprotective gene expression in renal medullary cells, which are routinely faced by high extracellular osmolalities. Recent data however indicate profound biological importance of the mammalian osmotic stress response in view of NFAT5 dependent gene regulation in non-renal tissues. In mononuclear cells and epithelial cells, NFAT5 stimulates the expression of various pro-inflammatory cytokines during elevated ambient tonicity. Accordingly, compared to plasma, the interstitial tonicity of lymphoid organs like spleen and thymus and that of liver is substantially hypertonic under physiological conditions. In addition, anisotonic disorders (hypernatremia, diabetes mellitus, dehydration) entail systemic hyperosmolality, and, in inflammatory disorders, the skin, intestine, and cornea are sites of local hyperosmolality. This article summarizes the current knowledge regarding systemic and local osmotic stress in anisotonic and inflammatory disorders in view of NFAT5 activation and regulation, and NFAT5 dependent cytokine production.

## INTRODUCTION

NFAT5 is the most recently identified member of the *Rel* family of transcription factors including NF-κB and nuclear factors of activated T cells (NFAT) 1-4 [1, 2]. NFAT5 was cloned independently by three groups in 1999 and was designated as tonicity-responsive enhancer binding protein (TonEBP) [3], NFAT5 [4], or osmotic response element binding protein (OREBP) [5] – In the following referred to as NFAT5. Based on amino acid sequence and structural analysis, NFAT5 shares about 40% sequence homology within the DNA binding domain with other members of the NFAT family [3, 4, 6]. In contrast to NFAT1-4 however, NFAT5 lacks a calcineurin-sensitive regulatory domain and is therefore not a target for calcineurin inhibitors. 

Whereas NFAT1-4 are only found in vertebrates, an ortholog of NFAT5 has been discovered in *Drosophila*, suggesting that the role of NFAT5 is not restricted to osmoregulatory processes in the kidney medulla in vertebrates, but is relevant to responses present in both vertebrates and invertebrates [7-9]. In mammalians, investigations addressing the function of NFAT5 initially primarily focused on the kidney medulla, since the cells of this kidney region are physiologically exposed to highly elevated interstitial osmolalities as a consequence of the operation of the renal concentrating mechanism [10]. In this kidney region, NFAT5 regulates the expression of various osmoprotective genes that are required for normal function and integrity of renal medullary cells [10-12]. These include transporters and synthetic enzymes mediating the intracellular accumulation of small, compatible organic osmolytes (i.e. inositol, sorbitol, betaine, taurine, glycerophosphocholin) [12]. By this mechanism, renal medulla-resident cells achieve osmotic equilibrium with the hyperosmotic extracellular space and thereby prevent cell shrinkage and apoptosis [12] (see below).

The observation that NFAT5 is expressed in virtually all tissues suggests that the function of NFAT5 is not limited to the renal medulla [13]. Several additional genes regulated by NFAT5 that are not directly involved in cellular osmoadaptation have been identified during the recent years (Table **1**). These include genes involved in embryogenesis and development, tumor metastasis, and hepatic detoxification enzymes, suggesting profound biological importance of NFAT5 distinct from osmoadaptation. Table **1** summarizes these novel findings, although this topic will not be an extensive subject of the present review.

In addition to the activation of conserved adaptive mechanisms to balance intra- and extracellular osmolalities, osmotic stress affects the function of the immune system. Hypertonicity stimulates the secretion of pro-inflammatory cytokines in immune cells and epithelial cells, and, intriguingly, several pathologies are associated with local osmotic stress within the cellular microenvironment and altered immune responses. These observations suggest that specific responses to hyperosmolality are integral part of the immune responses by stimulating pro-inflammatory cytokine production.

The following sections summarize the current knowledge regarding local osmotic stress in human disorders and the role of NFAT5 activation in view of inflammatory cytokine expression under these conditions. This is preceded by a brief overview of the role of NFAT5 in osmoadaptation and the regulation of NFAT5.

## ROLE OF NFAT5 IN OSMOADAPTATION

The normal plasma osmolality of 290-300 mosm/kg H_2_0 is tightly regulated by the release and action of vasopressin, and alterations in systemic osmolality entail severe consequences both at the cellular and systemic level [10, 12]. Dehydration and hence systemic hyperosmolality remain major challenges to land organisms because they evolved from the sea, where water is readily available. To adapt to fluctuations in water availability and intake, land-based mammalians, including humans, have developed an effective urinary concentrating mechanism, that allows adjustment of water excretion to the volume status of the body. As a consequence of the operation of the urinary concentrating mechanism, renal medullary cells are faced by extremely elevated extracellular osmolalities, which may reach 1,200 mosm/kg H_2_0 in humans and may exceed 6,000 mosm/kg H_2_O in desert rodents [25]. 

Extracellular hyperosmolality entails extraction of water from cells, which causes cell shrinkage, a rise in cellular ionic strength, an increase in macromolecular crowding, and alterations in subcellular architecture [10, 12, 26]. To overcome these potentially lethal conditions, osmotic stress stimulates the transcription of several genes involved in the intracellular accumulation of small organic osmolytes, such as sorbitol, *myo*-inositol, betaine, taurine, and others [27]. In contrast to electrolytes, organic osmolytes can be enriched intracellularly in the range of >500 mmoles/liter without detectable negative effects on cellular metabolism or function [25]. By this mechanism, cells exposed to significant osmotic stress achieve osmotic equilibrium with the extracellular space and therefore prevent the harmful effects of hypertonicity. For a comprehensive overview on genomic and cellular responses to osmotic stress, the reader is referred to recent reviews on this subject [10, 12, 26, 28].

## REGULATION OF NFAT5 ACTIVITY

NFAT5 target genes contain highly conserved sequences within their promoter region, known as *tonicity responsive enhancer (TonE)*, to which NFAT5 binds and stimulates the transcription of the respective target genes [28]. Notably, the NFAT1-4 cognate motiv 5'-(T/A/C)GGAA(A/G)-3' is encompassed within the NFAT5 recognition site 5'-TGGAAA(C/A/T)A(T/A)-3' [23]. Accordingly, NFAT5 has been demonstrated to bind to NFAT1-4 sites and to stimulate transcription, however with lower affinity than other NFAT members [29]. Although partial similarities in the core recognition sequence between NFAT1-4 and NFAT5 have been described, activation of NFAT5 differs in several aspects from that of NFAT1-4. While members of the NFAT1-4 family form heterodimers with additional transcription factors like AP-1, NFAT5-stimulated gene expression requires homodimer formation to bind to the respective consensus sites [4, 6]. In addition, nuclear translocation of NFAT5 is not regulated by the phosphatase calcineurin as in the case of NFAT1-4 [30].

Various mechanisms have been described for regulation of NFAT5 activity under hypertonic conditions. (i) Hypertonicity causes a rise in intracellular ionic strength, which is directly sensed by the N-terminal transactivation domain [31-33]. Accordingly, the activity of the latter directly correlates with with extracellular osmolality [31]. (ii) Several kinases have been implicated in NFAT5 phosphorylation and activation, the most important being p38, Fyn, ATM, and PKA, although the upstream activators are incompletely characterized [2, 34]. Furthermore, (iii) nuclear translocation and (iv) homodimer formation are required for NFAT5-dependent gene transcription during osmotic stress [35]. (v) In yeast and bacteria, a membrane associated osmosensor has been identified that signals to the homologue of mammalian p38 in response to hypertonicity [36, 37]. Accordingly, we have recently identified the EGF receptor as an osmosensitive transmembrane signal transducer that signals to p38 and stimulates NFAT5 transcriptional activity in response to hypertonicity in renal medullary cells [38, 39]. Whether this mechanism is also relevant for non-renal cells remains to be determined, however a recent study suggested that small G proteins and the nucleotide exchange factor Brx contribute to NFAT5 activation in lymphocytes exposed to hypertonicity [14]. Thus, membrane-associated signaling events in response to osmotic stress appear to be a common mechanism for NFAT5 activation in different cell types. (vi) Finally, in addition to the mechanisms described above, the abundance of NFAT5 increases under hypertonic conditions by elevated transcription of the NFAT5 gene and by increased stability of the respective mRNA [40, 41]. 

## ROLE OF NFAT5 IN LYMPHOID FUNCTION

The concept of isotonicity of body fluids and tissues (excluding the renal medulla) had to be revised by seminal studies by Go *et al*. [42]. The authors provided evidence that the interstitium of lymphoid organs like thymus and spleen (and also that of liver) is hyperosmolar in the range of 330-340 mosm/kg H_2_O compared to plasma with ~300 mosm/kg H_2_O [42]. In heterozygous NFAT5 knockout mice that are viable and show partial loss of function, the cellularity of thymus and the content of mature T and B cells is diminished by ~30%, and specific IgG production upon antigenic stimulation is significantly reduced [42]. These defects could be reproduced ex vivo in primary lymphoyctes from hypomorphic NFAT5 animals, which showed reduced proliferation and cytokine production under conditions of moderately elevated extracellular osmolality, which was a functional consequence of impaired osmoadaptation of lympoid cells in their local environment of lymphoid organs [42]. This notion if further supported by a recent study demonstrating that NFAT5 is required to prevent downregulation of cyclins and cell cycle arrest during osmotic stress [43]. Further support for a significant role of NFAT5 in immune responses stems from the observation that NFAT5 is induced in T cells upon T cell receptor activation [13] and that NFAT5 is involved in the regulation of B cell proliferation, differentiation, and immunoglobin production through induction of B cell activating factor (BAFF) [14]. These observations suggest that proper osmoadaptation is an essential component of normal lymphoid function and immune responses. Whether inhibition of NFAT5 might be useful in cases of overstimulation of the immune system like autoimmune disorders or transplant rejection remains to be determined.

## PATHOLOGIES ASSOCIATED WITH HYPEROSMOLALITY AND INFLAMMATION

During the recent years it has become increasingly clear that local or systemic hyperosmolality is evident during the course of various inflammatory disorders (Table **2**). Accordingly, a variety of major human pathologies is associated with hyperosmolality and inflammatory responses. In some conditions, a direct connection between cytokine secretion and NFAT5 activation has already been established (Table **3**), in others, it appears highly intriguing to speculate that NFAT5 plays a significant role in the initiation and progression of the disease process.

In addition to blood cells, epithelial cells play essential roles during the immune response after challenge with exogenous or endogenous stimuli in view of their ability to secrete various cytokines and chemokines [44-46]. Importantly, direct NFAT5-mediated osmotic regulation of TNF-α and lymphotoxin-β has been demonstrated in T cells [23], and IL-1, IL-6, and IL-18 contain putative NFAT5 consensus sites in their promoter region and are regulated in a NFAT5 dependent manner under hypertonic conditions (unpublished result). The following section summarizes the available knowledge on the role of NFAT5 in pathologies associated with local or systemic hyperosmolality.

### NFAT5 and Diabetic Microvascular Lesions

Up to 50% of patients with type 1 and type 2 diabetes will develop microvascular complications during the course of the disease, including diabetic neuropathy, retinopathy, and nephropathy [57]. It is generally accepted that chronic inflammation and dysregulation of the immune system play important roles in the development of microvascular lesions in diabetes. Hyperglycemia may result in significant plasma hyperosmolality in the range of 310-350 mosm/kg H_2_O, and postprandial hyperglycemia frequently occurs even when metabolic control is apparently good [49, 58, 59]. In addition, oscillatory hyperglycemia as routinely found in most patients, causes more acute increases in plasma concentrations of pro-inflammatory cytokines including IL-6, IL-18, and TNF-α, as compared to chronic hyperglycemia [60]. In support of the notion that hyperosmolality is linked to cytokine expression, exposure of human peripheral blood monocytic cells (PBMC) to hyperosmotic conditions (330-410 mosm/kg H_2_O) induces expression of IL-1 and IL-8 [61]. Indeed, increased DNA binding activity of NFAT5 to TonE is significantly increased in PBMC from patients with microvascular lesions compared with diabetic control subjects [62]. Since TNF-α is a direct NFAT5 target gene in immune cells [23] and IL-1, IL-6 and IL-18 contain NFAT5 binding sites in their promoter region (unpublished result), it is readily conceivable that NFAT5 plays a causal role in inflammatory processes as observed in diabetic microvascular lesions. These observations indicate that persistent or intermittent hyperosmolality, as present in diabetic subjects, is sufficient for activation of NFAT5 and likely inflammatory cytokine secretion.

It is well established that hyperglycemia results in increased glucose metabolization through the polyol pathway [63], which has been linked to abnormalities found in diabetic microvascular disease. In the first rate-limiting step, glucose is reduced to sorbitol by the enzyme aldose reductase (AR) by the usage of NADPH [64]. In the second step, sorbitol is oxidized to fructose by sorbitol dehydrogenase. This metabolic pathway entails reduced cellular NADPH and consequently gluthatione levels, which may predispose to oxidative stress-induced damage [65]. In addition, elevated fructose levels increase the formation of advanced glycosylation end products [65]. The notion that NFAT5 is causally linked to these events is supported by the observation that AR is a prototypical NFAT5 target gene [66], and that NFAT5 shows increased DNA binding activity to its consensus sequence in PBMC from patients with diabetic microvascular complications compared with diabetic control subjects under hyperglycemic conditions [62]. Furthermore, enhanced expression of AR is observed in peripheral nerve and renal glomeruli in diabetic patients, and the degree of expression correlates with the disease score [67].

Data regarding NFAT5 inhibition are lacking so far, however, orally available AR inhibitors have been developed over the recent 25 years and tested in clinical trials [55]. The results were however mixed and several substances had to been withdrawn from human use because of adverse side effects. Currently, only one compound (epalrestat) is approved in Japan. Future work is required to address the question whether inhibition of NFAT5 might be suitable to ameliorate diabetic microvascular complications. We are currently testing this exciting hypothesis in conditional NFAT5 knockout mice.

### Role of NFAT5 in Blood Pressure Regulation and Sodium-Sensitive Hypertension

Salt sensitive hypertension describes a reproducible condition between salt intake and blood pressure, and the development of arterial hypertension [68]. During the recent decade, the concept of isotonicity of body fluids (i.e. the plasma and tissue interstitium) had however to be revised. Studies by the group of Titze convincingly demonstrated that sodium can be accumulated in the interstitium of the skin without commensurate water retention, and this skin sodium storage is paralleled by increased polymerization and sulfation of skin glycosaminoglycans, thereby increasing their ability for sodium storage [69-71]. Indeed, a high salt diet in rats causes hypertonic sodium storage within the interstitium of the skin, and this condition correlates with the development of arterial hypertension [24]. The ensuing local osmotic stress is sensed by skin macrophages, which activate the NFAT5 pathway.

We could identify vascular endothelial growth factor (VEGF)-C as a direct NFAT5 target gene in macrophages. Accordingly, the synthesis of VEGF-C increases in a NFAT5-dependent manner in skin macrophages of rats fed a high salt diet and in macrophages exposed to hypertonicity *in vitro* [24]. In consequence, VEGF-C induces hyperplasia and increased density of the skin lymphcapillary network, which buffers hypertension. Conversely, blocking NFAT5 or VEGF-C signalling aggravates hypertension. Thus, NFAT5–VEGF-C signalling in skin macrophages is a major determinant of extracellular volume and hence blood pressure homeostasis under conditions of high salt intake [24].

### Inflammatory Bowel Disease

Human inflammatory bowel disease (IBD), the most important entities being Crohn's disease and ulcerative colitis, are chronic and relapsing inflammatory conditions that result from chronic dysregulation of the mucosal immune system in the gastrointestinal tract [72].

Elevated levels of pro-inflammatory cytokines, including IL-1, IL-6, IL-18, and TNF-α are detected in active IBD, and correlate with the severity of inflammation, the degree of mucosal permeability leak, and leukocyte infiltration [73-75]. The major source of inflammatory cytokines are colonic epithelial cells and possibly infiltrating immune cells. Intriguingly, patients with Crohn's disease present with substantially higher osmolality of the faecal fluid compared to control subjects (~490 vs. ~340 mosmol/kg H_2_O), and faecal hyperosmolality correlates with the histological disease score [53, 54]. Given the fact that colonic epithelial cells express aquaporins in their apical membrane, luminal hyperosmolality is expected to translate into intracellular osmotic stress and hence NFAT5 activation, which is abundantly expressed in colonic epithelial cells [21]. Although a direct causal link between the inflammatory process and NFAT5 activation is currently missing, the availability of mouse models with characteristics similar to human IBD and the use of conditional NFAT5 knockout mice will hopefully shed further light on this subject. 

### Miscellaneous

Diverse other conditions are characterized by local osmotic stress, which correlates with the secretion of pro-inflammatory cytokines by the respective cell type, although a direct functional role of NFAT5 has not been demonstrated yet (See also Table **2**).

Exercise-induced bronchoconstriction and inflammation occurs frequently in patients with bronchial asthma or chronic obstructive pulmonary disease with a delay of 2-13 hours after exercise [76, 77]. Evaporation of water across airway epithelia results in substantial local hyperosmolality of the airway mucosa (Table **2**) [78]. Accordingly, human respiratory epithelia shrink after apical exposure to hypertonic saline, which causes epithelial cell activation, along with secretion of IL-8 and RANTES [79-81]. The latter effects require p38 MAP kinase activity, which is an established upstream activator of NFAT5 [81, 82]. Whether NFAT5 plays a causal role in this process is currently unknown, however, both IL-8 and RANTES harbor NFAT5 target sequences in their promoter region (unpublished result).

Hyperosmolality of the tear film is regarded as the major factor in the pathogenesis of corneal inflammation in dry eye syndrome, which is a highly prevalent condition affecting 10-20% of adults [47, 83-86]. Osmolalities up to 365 mosm/kg H_2_O have been reported in patients with sicca syndrome, while it was 280-300 mosm/kg H_2_O in normal subjects [47] (Table **2**). Accordingly local osmotic stress is associated with elevated concentrations of IL-1, IL-6, TNF-α, and others in the tear film and induction of the respective mRNAs in corneal and conjunctival epithelia, and cytokine levels correlate with clinical parameters [87, 88].

Hypernatremia is a serious electrolyte disorder leading to substantially elevated plasma osmolality, representing a strong and independent risk factor for mortality in hospitalized patients [51, 89]. Hypernatremia may result from various causes, including faecal fluid losses, diabetes insipidus, diuretic use, burns, inadequate hypertonic fluid therapy, dehydration after exercise, heat stroke, and others. Due to the relative excess of sodium to water in the extracellular fluid, plasma osmolalities may exceed 340 mosm/kg H_2_O [89]. Severe hypernatremia is associated with increased circulating levels of inflammatory mediators including IL-6, IL-10, and TNF-α, even in the absence of an infective focus [89, 90]. Given the fact that immune cells produce these cytokines in response to osmotic stress, it is readily conceivable that this event is mediated by activation of NFAT5.

## CONCLUDING REMARKS

Recent studies on NFAT5 function primarily focused on osmoadaptation of renal medullary cells, however emerging evidence suggests profound biological importance of NFAT5 activation in view of local hyperosmolality associated with inflammation as found in many human pathologies. Further investigation of this hypothesis in NFAT5 knockout mice has been largely hampered by dramatically reduced embryonic viability and a high degree of early perinatal lethality in homozygeous NFAT5-deficient mice [42]. To overcome this limitation, we have recently generated conditional NFAT5 knockout mice, which will hopefully shed further light on the function of NFAT5 in the course of inflammatory disorders. In addition, future experiments are required to clarify whether inhibition of NFAT5 might be a suitable target for treatment of inflammatory pathologies associated with osmotic stress.

## Figures and Tables

**Table 1:** NFAT5 Target Genes not Directly Involved in Osmoadaptation

Target gene	Cell type	Function	Refs.
B cell activating factor (BAFF)	B lymphocytes	B lymphocyte proliferation, differentiation, and IgG production	[14]
beta1,3-Glucuronosyltransferase-I (GlcAT-I)	Nucleus pulposus cells (intervetebral disk)	Glycosaminoglycan synthesis	[15]
Cyclooxygenase-2 (COX-2)	Renal medullary cells	Prostaglandin synthesis	[16]
Cytochrome p450 2E1, 3A	Hepatocytes	Drug metabolism	[17,18]
HIV replication	Macrophages	Binding of NFAT5 to LTR required	[19]
S100A4/metastasin	Cancer cells	Tumor metastasis	[20,21]
Myocyte differentiation	Myoblasts	Cyr61	[22]
TNF-α, lymphotoxin-β	T lymphocytes	Immune responses	[23]
VEGF-C	Macrophages	Lymphangiogenesis	[24]

**Table 2:** Pathologies Associated with Local Hypertonicity and Inflammation

Disorder	Osmolality (mosm/kg H_2_O)	Refs.
Corneal inflammation in dry eye syndrome	330-365 (tear film)	[47]
Diabetes mellitus	310-350 (serum)	[48, 49]
Exercise-induced airway inflammation in obstructive pulmonary disorders	350 (bronchial fluid)	[50]
Hypernatremia	340 (serum)	[51,52]
Inflammatory bowel disease	490 (faeces)	[53, 54]

**Table 3:** Systemic Disorders with Proven Involvement of NFAT5 Target Genes

Condition/Disorder	Target gene	Refs.
Diabetic microvascular lesions	Aldose reductase Inflammatory cytokines?	[55]
Adaptive immunity	Genes involved in osmoadaptation Immune modulatory cytokines B cell activating factor (BAFF)	[14, 23, 42]
Salt-sensitive hypertension	VEGF-C	[24, 56]
